# Slowing disease accumulation but persistent complexity: population-level multimorbidity dynamics in old age

**DOI:** 10.1093/ageing/afag268

**Published:** 2026-09-11

**Authors:** Yuge Zhang, Marcus Ebeling, Katharina Schmidt-Mende, Sven Drefahl, Karin Modig

**Affiliations:** Institute of Environmental Medicine, Karolinska Institutet, Stockholm, Sweden; Research Group Medical Demography, Max Planck Institute for Demographic Research, Rostock, Germany; Department of Neurobiology, Care Sciences and Society, Karolinska Institutet, Huddinge, Sweden; Academic Primary Health Care Centre, Stockholm, Sweden; Demography Unit, Department of Sociology, Stockholm University, Stockholm, Sweden; Institute of Environmental Medicine, Karolinska Institutet, Stockholm, Sweden

**Keywords:** population dynamics, disease burden, multimorbidity, old age, disease trajectories, older people

## Abstract

**Background:**

Most research on multimorbidity has focused on individual disease trajectories, with less attention to how disease accumulation evolves at the population level as cohorts age. Understanding these dynamics is essential for planning care in ageing populations. This study aimed to characterize population-level trajectories of disease accumulation between ages 70 and 100 and quantify the contributions of survivorship, proximity to death and disease composition.

**Methods:**

Using nationwide register data from Sweden, we examined disease accumulation between ages 70 and 100 amongst all individuals born in 1920–1922 who survived to age 70, assessing the roles of survivorship, proximity to death, disease composition.

**Results:**

Multimorbidity increased substantially between ages 70 and 100. Disease accumulation accelerated up to age 80, stabilized between ages 80 and 90, and decelerated thereafter, although both disease burden and complexity continued to increase into extreme old age. By age 90, the mean number of diseases was 3.9 in men and 3.5 in women, and more than half of individuals aged ≥95 had five or more diseases. Most accumulation occurred during non-terminal years of life. Cardiovascular disease was the dominant contributor throughout, whilst musculoskeletal and neurosensorial diseases became increasingly important at advanced ages.

**Conclusion:**

Multimorbidity continues to accumulate and diversify well into extreme old age. Although the pace of accumulation slows after age 90, disease burden and complexity continue to increase. These findings highlight that multimorbidity in late life is primarily driven during non-terminal years and increasingly characterized by complex, cross-system disease patterns, with important implications for geriatric care.

## Key Points

- Disease accumulation accelerated up to age 80, stabilized between ages 80 and 90, and decelerated thereafter.
- Disease accumulation in late life was primarily driven during non-terminal years.
- Proximity to death increasingly contributed to population disease burden at older ages.
- Both disease burden and complexity continued to increase into extreme old age, with sex difference in disease composition.
- Cardiovascular disease remained the dominant contributor of multimorbidity across ages

## Introduction

Most research on multimorbidity—defined as the coexistence of two or more chronic diseases [1]—has examined how individuals accumulate diseases over time [2, 3]. Less attention has been paid to how disease accumulation evolves at the population level as cohorts age and the composition of the surviving population changes. This population perspective captures the combined effects of disease accumulation, mortality and selective survival on the overall burden of multimorbidity and as such, it constitutes a central force shaping population health. Understanding how disease accumulation evolves with age, its underlying drivers and its consequences for the overall disease spectrum is therefore crucial for public health and geriatric medicine, particularly in the context of population ageing and increasing longevity.

Population-level disease accumulation is shaped by two fundamental processes: age-specific disease incidence and survival, which determines who remains at risk and contributes to the observed burden. For many chronic conditions, incidence rises steeply with age. Evidence from studies of individual diseases (e.g. stroke, myocardial infarction and many cancers) suggests that incidence rates often peak near average life expectancy (around age 85 in high-income settings) and may subsequently plateau or decline [4]. However, these patterns are largely derived from single-disease analyses and may reflect competing risks rather than broader changes in overall morbidity.

At the same time, survival differs across diseases. This makes the type of diseases accumulated the main source of selective survival, whereby individuals reaching advanced ages are a non-random subset with respect to their morbidity profiles [4, 5]. Such selection mechanisms likely interact with both the type and extent of diseases accumulated, and thus, potentially alter the shape of population-level disease accumulation at older ages. In addition, a substantial body of evidence characterizes the end of life as a period of intensified disease onset and diagnosis. The process of dying itself may therefore represent an important driver of population-level disease accumulation.

Taken together, these mechanisms suggest that disease accumulation at the population level may follow different trajectories in advanced age. On the one hand, increasing incidence would be expected to sustain continued growth in morbidity. On the other hand, mortality and selective survival may alter the distribution and combinations of diseases amongst individuals surviving to older ages, potentially leading to an apparent deceleration or plateau in population-level disease accumulation [6, 7]. However, empirical evidence on these patterns remains limited and is largely based on single-disease analyses.

This study examines how disease accumulation evolves within a nationwide birth cohort, how mortality and survivorship shape this process, and which diseases contribute most to its increase at older ages. Using nationwide register data, we analyse disease accumulation and combinations of diseases between ages 70 and 100, and examine how these patterns change as the number of surviving individuals declines. By combining population-level and age-by-age perspectives on multimorbidity, this study aims to characterize how disease burden evolves throughout advanced age and to identify patterns of disease accumulation across the oldest decades of life.

## Methods

### Study population

The study was based on nationwide Swedish administrative registers linked using the unique personal identity number assigned to all Swedish residents. The Total Population Register provided information on birth year, sex, residence, emigration and vital status [8]. All individuals born in 1920, 1921 and 1922, who survived to age 70 and resided in Sweden until death were included. Using historical administrative health care records, individuals were followed from age 70 onwards, beginning 1 January 1990, until death or 31 December 2022 (maximum attained age 100). To define prevalent conditions at baseline, a 3-year look back period (ages 67–69; calendar years 1987–1989) was used to establish each individual’s disease profile at age 70. As the cohort aged, the observation period extended correspondingly amongst survivors.

### Measures of disease

Diseases were identified from inpatient and outpatient care records in the National Patient Register using International Classification of Diseases (ICD) codes recorded as either primary or secondary diagnoses [9, 10]. Nationwide inpatient data have been available since 1987 and specialist outpatient data since 2001 and include essentially all specialist care in Sweden even if the provider is privately operated. See Appendix 1 in the Supplementary Data section for the details of the registers, data coverage and linkage procedures. We used a previously developed list of chronic conditions for multimorbidity research in older populations [11, 12] adopting the same disease groups whilst adding five additional age-related chronic conditions [13–17]. The final disease list comprised 40 chronic conditions grouped into ten disease categories: anaemia, cardiovascular, digestive, endocrine, malignancy, neuropsychiatric, musculoskeletal, neurosensorial, respiratory and urological diseases. See Appendix 2 in the Supplementary Data for the full details of included diseases. Acute conditions, including infectious diseases, were not included.

Disease patterns were described at ages 70, 80, 90 and 100 years. For each disease, the date of the first recorded diagnosis was used to define disease onset. Because all conditions included in the analyses were considered chronic, diseases were assumed to persist thereafter.

### Statistical analysis

We first described changes in population multimorbidity across age by calculating the age-specific distribution of individuals with 0, 1, 2, 3, 4 and ≥ 5 chronic diseases. Population-level disease accumulation was then quantified by calculating the age-specific mean number of diseases from age 70 onwards. This measure provides an intuitive summary of disease accumulation at the population level, is directly comparable across ages, and can be decomposed into contributions from disease accumulation and survival. We additionally calculated age-to-age changes in the mean number of diseases to characterize the speed of disease accumulation across later life.

To better understand the mechanisms underlying changes in disease accumulation, in particular how much of disease accumulation is driven by individuals in close proximity to death, we conducted two decomposition analyses. First, we quantified the extent to which changes in the age-specific mean number of diseases ($\overline{C_x}$) were driven by (i) disease accumulation amongst long-term survivors, (ii) disease accumulation amongst individuals in close proximity to death, and (iii) the increasing proportion of individuals approaching the end of life with advancing age. For these analyses, individuals were classified as being in close proximity to death if they died within 2 years after the age under consideration. Individuals who survived for >=2 years beyond that age were classified as long-term survivors.

The age-specific mean number of diseases, $\overline{C_x}$ was expressed as a function of the mean number of diseases amongst long-term survivors ($\overline{S_x}$), the mean number of diseases amongst individuals in close proximity to death ($\overline{D_x}$), and the proportion of individuals in close proximity to death (${d}_x$), which is $\overline{C_x}=\overline{S_x}+{d}_x\left(\overline{D_x}-\overline{S_x}\right)$. This decomposition partitions the change in the average number of diseases between two ages into three components: (i) changes in disease burden amongst long-term survivors, (ii) changes in disease burden amongst individuals in close proximity to death and (iii) changes in the proportion of the population in close proximity to death. To estimate the contribution of each component, we applied a general decomposition algorithm that empirically approximates partial derivatives [18]. Contributions were quantified these contributions over the full age range (70–100 years) and separately for the age intervals 70–80, 80–90 and 90–100 years.

In the second decomposition analysis, we quantified how changes in the average number of chronic diseases within each disease group contributed to changes in the overall age-specific mean number of diseases ($\overline{C_x}$). Because ($\overline{C_x}$) equals the sum of the mean number of diseases across all disease groups, each disease group’s contribution could be quantified by summation over the age range of interest. Contributions were estimated for ages 70–100 years overall and separately for the age intervals 70–80, 80–90 and 90–100 years.

Finally, we characterized the prevalence and combinations of disease groups at ages 70, 80, 90 and 100 in men and women. Diseases were considered chronic and therefore remained present after their first recorded diagnosis. Accordingly, once a disease group was identified, it was assumed to persist at all subsequent ages. Because cardiovascular diseases (CVD) were the most prevalent disease group, we additionally examined the prevalence and combinations of individual CVD. To further elucidate age-related dynamics in multimorbidity complexity, we examined the prevalence and combinations of specific disease groups, as well as individual CVD, separately amongst long-term survivors and individuals in close proximity to death.

All analyses were conducted using SAS 9.4 (SAS Institute Inc., Cary, NC, USA) and R (version 4.4.1).

## Results

Of the 197 160 men and 186 262 women born between 1920 and 1922, 64.2% of men (126 630) and 79.2% of women (147 478) survived to age 70, were residing in Sweden, and were eligible for inclusion. See Appendix 3 in the Supplementary Data section for the details of mortality selection of the cohorts. Overall, disease accumulation accelerated between ages 70 and 80, remained relatively stable until approximately age 90, and then slowed thereafter, whilst the complexity of multimorbidity continued to increase throughout old age.

### Disease accumulation over age

**Figure 1:**
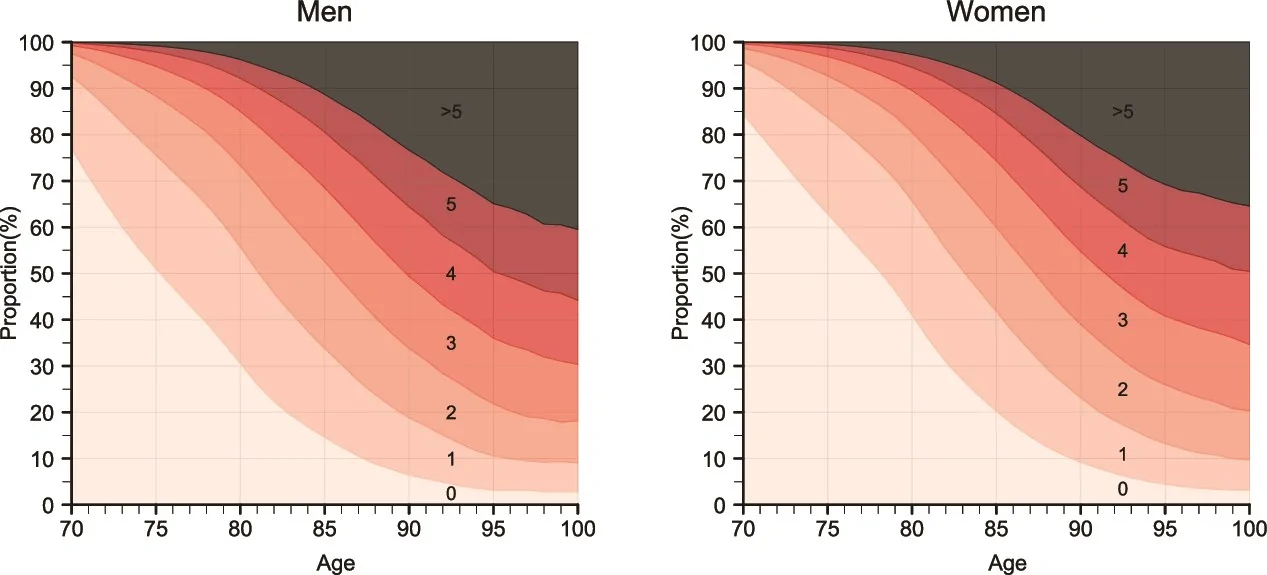
Proportion of individuals with 0 to >5 diseases, ages 70 to 100, men and women, birth cohorts 1920-1922, Sweden.

Figure 1 shows the distribution of individuals with 0 to ≥5 diseases between ages 70 and 100, stratified by sex. By age 85, ~65% of men and 58% of women had developed multimorbidity (defined as at least two diseases). The proportion with one to two diseases increased sharply until around age 90 and then stabilized. In contrast, the proportion with five or more diseases rose modestly before age 85 but increased steeply thereafter. Around age 85, having five or more diseases gradually became the most common category. After age 95, >50% of men and 45% of women had five or more diseases, underscoring the persistence of multimorbidity into the oldest ages.

**Figure 2:**
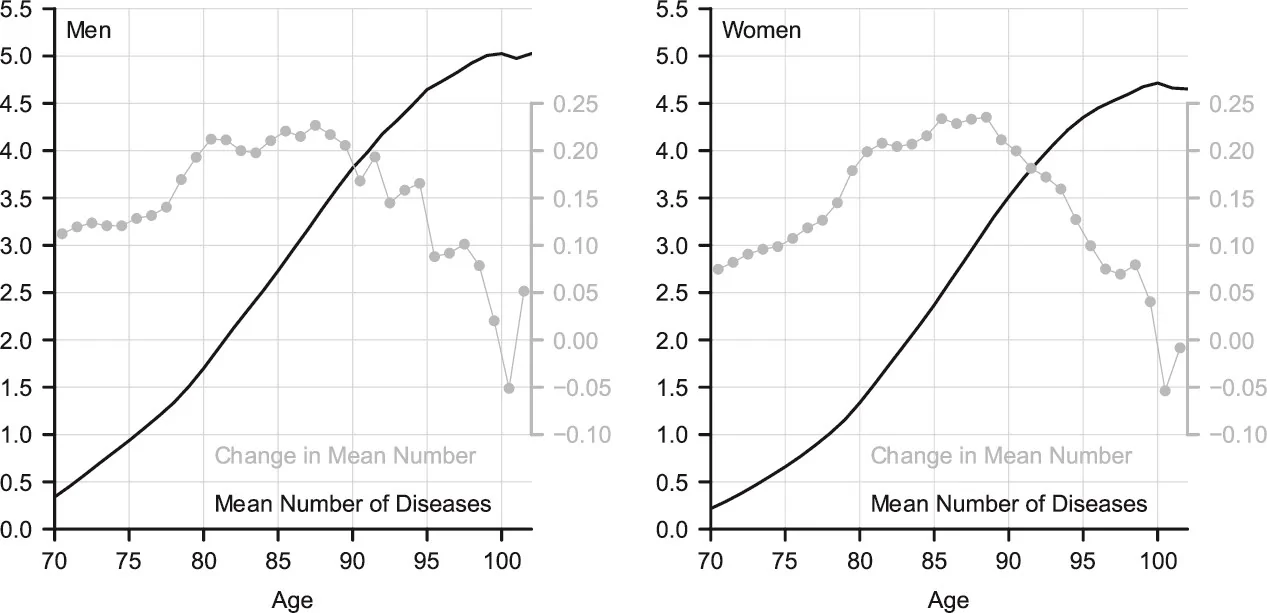
Mean number of diseases (left Y-axis) and age-to-age changes (right Y-axis), ages 70 to 100, men and women, birth cohorts 1920-1922, Sweden. Note: The mean number of diseases was calculated from 40 chronic conditions grouped into ten disease categories: anaemia, cardiovascular, digestive, endocrine, malignancy, neuropsychiatric, musculoskeletal, neurosensorial, respiratory, and urological diseases. Age to-age changes represent the absolute change in the average number of diseases between consecutive ages.

Figure 2 illustrates the mean number of diseases and its age-to-age changes from age 70 to 100. At all ages, men had a higher mean number of diseases than women. The mean increased from ~0.25 (women) and 0.4 (men) at age 70 to 3.5 (women) and 3.9 (men) at age 90. Thereafter, the increase was more modest, approaching a plateau at ~4.6 (women) and 5.0 (men) by age 97. This deceleration is also reflected in the age-to-age changes, which followed a hump-shaped pattern: accumulation accelerated until age 80, stayed almost constant between ages 80 and 90 (peaking at ~0.2 additional diseases per year), and then decelerated after age 90.

**Figure 3:**
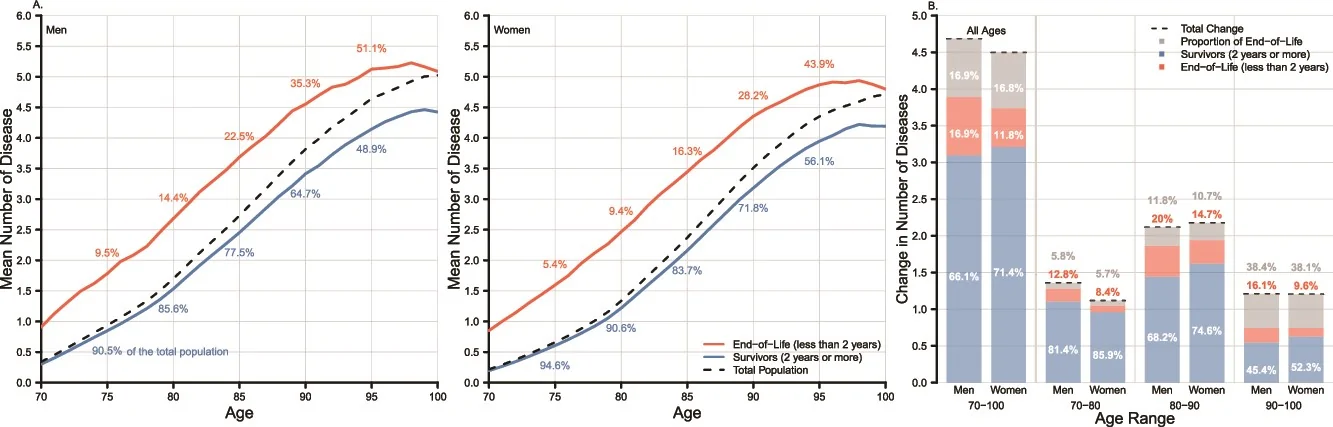
Mean number of diseases by remaining lifespan (A) and decomposition of changes in mean number of diseases (B), ages 70 to 100, men and women birth cohorts 1920-1922, Sweden. Note: In panel A, lines show the age-specific mean number of diseases in the total population and in subgroups defined by remaining lifespan: total population (black), individuals in close proximity to death, defined as dying within two years after the age under consideration (red), and long-term survivors, defined as surviving >=2 years beyond that age (blue). Percentages indicate the age-specific proportion of the total population belonging to each subgroup. In panel B, bars show the absolute contribution of each component to changes in the mean number of diseases: changes among individuals in close proximity to death (red), changes among long-term survivors (blue), and changes in the proportion of individuals in close proximity to death (grey). Bar labels indicate the relative contribution of each component to the total change.

Figure 3 panel A presents disease accumulation in relation to survival and proximity to death. It shows that individuals within 2 years of death consistently had a higher disease burden than long-term survivors. As the proportion of individuals approaching death increased with age, their contribution to the population mean became progressively larger, particularly after age 90.

Panel B shows the contributions to disease accumulation from survivors and individuals near the end of life. Across the full age range, approximately two-thirds of the increase in disease burden was attributable to survivors, whilst individuals close to death accounted for 17% (men) and 12% (women) of the increase in the mean number of diseases between ages 70 and 100. The contribution of the shifting population composition towards a greater proportion of individuals near the end of life exceeded the contribution of disease accumulation within that group. Between ages 70 and 100, changes in population composition accounted for 17% of the overall increase, rising to nearly 40% of the increase observed between ages 90 and 100.

Appendix 4 in the Supplementary Data section shows the disease groups contributing most to the increase in disease burden. In both sexes, CVD accounted for nearly 40% of total accumulation between ages 70 and 100, although their relative contribution declined with age as other groups became more prominent. Women showed a substantially higher contribution from musculoskeletal diseases, whereas urological diseases contributed markedly less compared with men. Neurosensorial diseases showed the strongest increase across the three age groups.

### Disease patterns and combinations

**Figure 4:**
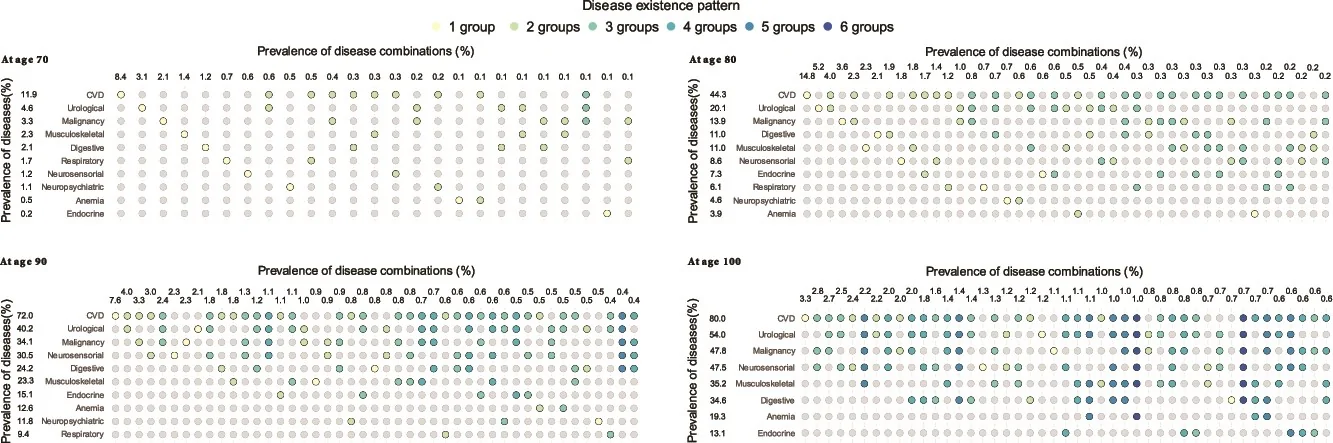
Disease combinations in men at age 70, 80, 90 and 100, birth cohorts 1920 to 1922, Sweden. Notes: The x-axis shows the prevalence of the 45 most common disease combinations, ranked in descending order. Disease combinations are represented by connected dots, with colors indicating the number of disease groups involved (single disease group: light yellow; 2 disease groups: yellow-green; 3: green-cyan; 4: cyan; 5: blue-cyan; 6: blue). Only disease combinations with a prevalence of at least 0.1% are shown.The rows labelled "Prevalence of diseases (%)" show the overall prevalence of each disease group, irrespective of whether it occurs alone or in combination with other disease groups. The dot matrix and the corresponding "Prevalence of disease combinations (%)" columns show the prevalence of specific disease combinations.Example: At age 70, 11.9% of men had cardiovascular disease (CVD), regardless of whether it occurred alone or together with other disease groups. 8.4% had CVD only, whereas 0.6% had both CVD and urological disease, as indicated by the connected dots.

**Figure 5:**
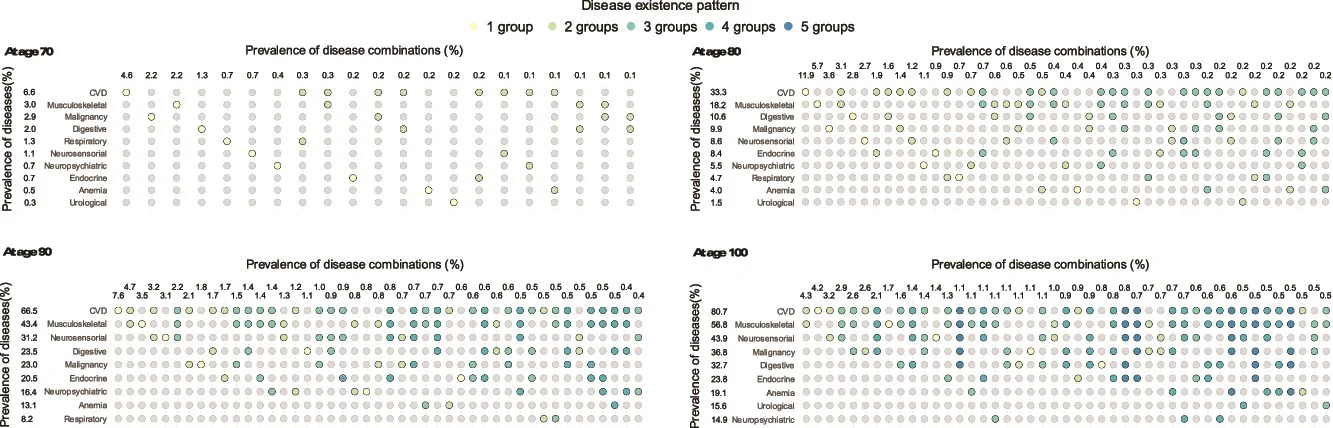
Disease combinations in women at age 70, 80, 90 and 100, birth cohorts 1920 to 1922, Sweden. Notes: The x-axis shows the prevalence of the 45 most common disease combinations, ranked in descending order. Disease combinations are represented by connected dots, with colors indicating the number of disease groups involved (single disease group: light yellow; 2 disease groups: yellow-green; 3: green-cyan; 4: cyan; 5: blue-cyan). Only disease combinations with a prevalence of at least 0.1% are shown.The rows labelled "Prevalence of diseases (%)" show the overall prevalence of each disease group, irrespective of whether it occurs alone or in combination with other disease groups. The dot matrix and the corresponding "Prevalence of disease combinations (%)" columns show the prevalence of specific disease combinations.Example: At age 70, 6.6% of women had cardiovascular disease (CVD), regardless of whether it occurred alone or together with other disease groups. 4.6% had CVD only, whereas 0.3% had both CVD and respiratory disease, as indicated by the connected dots.

Figures 4 and 5 illustrate how disease combinations evolve with age. Across all ages, CVD was the most prevalent disease group in both sexes. Amongst men, CVD, urological diseases and malignancies dominated, whereas women more often presented with CVD and musculoskeletal diseases, with neurosensorial conditions becoming increasingly prominent at older ages. Disease patterns were characterized by increasingly diverse combinations involving multiple disease groups, whilst some disease groups, such as neuropsychiatric and respiratory diseases, became less common at the oldest ages. Ischemic heart disease predominated at younger old ages, whereas hypertension became increasingly dominant at the oldest ages. Multimorbidity within the cardiovascular group became common from around age 80, with increasingly complex combinations observed at older ages. See Appendices 7 and 8 in the Supplementary Data section for the details of disease patterns within the cardiovascular group.

Complementary analyses excluding individuals close to death revealed similar patterns of disease combination but consistently lower overall prevalence (Appendices 5 and 6 in the Supplementary Data section).

### Specific combinations of cardiovascular diseases

At ages 70 and 80, ischemic heart disease was the most prevalent cardiovascular condition in both sexes, whereas hypertension predominated at ages 90 and 100 (Appendices 7 and 8 in the Supplementary Data section). The prevalence of most cardiovascular conditions increased sharply up to age 90 and then stabilized, whilst hypertension continued to rise into the oldest ages. Across nearly all ages, men had higher prevalence of ischemic heart disease, cerebrovascular disease and heart failure, whereas hypertension was more common amongst women.

Multimorbidity within the CVD group became the norm from around age 80. Whilst combinations of two or three conditions were most common, four or more complex disease combinations became increasingly frequent with age. Typical combinations included ischemic heart disease with heart failure in men, and hypertension combined with cerebrovascular or ischemic heart disease in women.

At more advanced ages, the number of distinct combinations increased, although their individual prevalence remained low. Analyses excluding individuals close to death showed similar patterns but consistently lower overall prevalence (Appendices 9 and 10 in the Supplementary Data section), suggesting selective survival of individuals with a lower burden of severe cardiovascular conditions. In this group, ischemic and cerebrovascular diseases were less common at age 100 than at age 90, whereas hypertension, atrial fibrillation and heart failure continued to increase. Notably, increasing complexity at older ages reflected a higher proportion of individuals with existing combinations rather than the emergence of new patterns.

## Discussion

In this population-based birth cohort study, we examined how multimorbidity evolves at the population level as cohorts age. We found that the rate of disease accumulation peaked between ages 80 and 90. This peak was preceded by accelerating accumulation before age 80, followed by deceleration after age 90. Importantly, this deceleration did not reflect stabilization of morbidity. Although the rate of accumulation slowed, both disease prevalence and the complexity of disease combinations continued to increase, indicating ongoing restructuring of multimorbidity at advanced ages. Overall, population-level disease accumulation was primarily shaped during non-terminal years of life, with distinct phases of change across age and shifting contributions from underlying disease groups.

The observed acceleration-deceleration pattern may reflect the changing balance between disease incidence and mortality with advancing age. Between ages 70 and 80, mortality levels remain relatively low, and disease profiles are shaped predominantly by increasing incidence. After age 80, mortality likely plays a larger role in shaping population disease profiles through selective survival. Disease accumulation would probably increase even more rapidly in the absence of this mortality selection, resulting in a dynamic balance between rising age-specific incidence rates and increasing mortality risk. At the same time, the composition of disease burden changes, shifting from more acute and fatal conditions towards chronic and survivable disorders [19]. Although diagnostic saturation at extreme ages may also contribute to the observed deceleration [20], the patterns are unlikely to be fully explained by this mechanism given the broad range of disease groups included. For example, the declining prevalence of some conditions, such as neuropsychiatric diseases amongst centenarians, more likely reflects selective survival than reduced incidence [21].

A key finding was the dominant role of CVD across all ages, both as an isolated condition and as a central component of multimorbidity, consistent with previous research [22, 23]. Although the importance of CVD persisted with age, the structure of disease combinations evolved substantially. Within the CVD group, the dominant combinations appeared relatively stable at advanced ages, suggesting predictable clinical trajectories [24]. In contrast, increasing clustering across disease groups, particularly involving musculoskeletal, neurosensorial and malignant conditions, drove the continued expansion of multimorbidity complexity.

Beyond the number of conditions, late-life multimorbidity was characterized by the emergence of increasingly complex disease combinations. By ages 90 and 100, combinations spanning four or more disease groups became increasingly prevalent. These findings suggest that multimorbidity in extreme old age is increasingly characterized by complex combinations of diseases spanning multiple disease groups, rather than by the accumulation of isolated conditions.

Although overall patterns were broadly similar in men and women, differences in disease composition were observed. Women more frequently exhibited combinations involving musculoskeletal conditions, whereas men showed greater clustering of cardiovascular, urological and malignant diseases. These patterns likely reflect sex-specific biological risks, hormonal influences and survival selection [25, 26]. The higher overall burden of multimorbidity observed amongst men contrasts with findings from several previous studies and may partly reflect our reliance on specialist healthcare data, which are likely to undercapture chronic conditions managed primarily in primary care, several of which are more common amongst women. Conversely, CVD, the largest contributor to multimorbidity in our cohort are generally well captured in hospital registers and were substantially more prevalent amongst men. Consistent with previous findings, men accumulated diseases earlier and more rapidly, whereas women survived longer despite a higher burden of multimorbidity [27].

Our findings refine interpretations of late-life plateaus in disease incidence and mortality. Although mortality selection increasingly shapes population composition at advanced ages, it does not lead to stabilization of total morbidity. Survivors at extreme ages represent a relatively resilient group, but resilience does not imply low disease burden. Rather, it appears compatible with continued accumulation and increasing complexity of multimorbidity. As life expectancy continues to rise, health systems should anticipate not only larger older populations, but also populations with increasingly complex disease profiles, requiring integrated and flexible models of care.

Strengths of this study include nationwide longitudinal register data covering an entire birth cohort, enabling detailed age-by-age analyses of multimorbidity from age 70 to 100 years. The complete population coverage, long follow-up and decomposition approach allowed us to disentangle the contributions of disease accumulation, proximity to death and population composition to multimorbidity trajectories in later life. However, some limitations should be acknowledged. Disease ascertainment relied on diagnoses recorded in specialist inpatient and outpatient care and did not include primary care data. Consequently, chronic conditions managed predominantly in primary care, such as hypertension and diabetes, are likely to be underascertained or recorded later in the disease course [28]. Although this may affect the estimated prevalence of some individual conditions, it is less likely to materially influence the age-related patterns of disease accumulation, which were the primary focus of this study. Moreover, our analyses describe the accumulation of clinically diagnosed chronic diseases and therefore do not capture the underlying biological processes of ageing. Future research should therefore integrate biomarkers and primary care data to better understand the relationship between clinical diagnoses and biological ageing.

## Conclusion

Multimorbidity continues to accumulate and diversify well beyond the average lifespan. Although the pace of accumulation slows after age 90, both overall disease burden and the complexity of disease combinations continue to increase into extreme old age. CVD play a central role in this process, with sex-specific differences shaping both timing and disease composition. More broadly, our findings indicate that disease accumulation occurs largely during non-terminal years of life, highlighting potential opportunities for prevention and treatment.

## Supplementary Material

aa-26-1608-File007_afag268

## Research Data Transparency and Availability

The individual level data underlying this study cannot be shared publicly because of the General Data Protection Regulation in Sweden. Access to the data and statistical code can be permitted to external researchers after ethical vetting and establishment of a collaboration agreement. Contact the corresponding author for questions about data sharing.
