# Supplementary material for: Slowing disease accumulation but persistent complexity: population-level multimorbidity dynamics in old age
Source: Age Ageing. 2026 Sep 11;55(9):afag268. doi: 10.1093/ageing/afag268 (PMC13565138; doi:10.1093/ageing/afag268)
Supplement: Supplementary file 1 [file aa-26-1608-file007_afag268.docx]

**Slowing disease accumulation but persistent complexity: population-level multimorbidity dynamics in old age**

Contents

[Appendix 1 Description of the registers included in current study	2](#_Toc237509328)

[Appendix 2 Swedish ICD9 and ICD10 codes used to identify diseases	3](#_Toc237509329)

[Appendix 3 Mortality selection of the cohorts 1920-1922, Sweden.	5](#_Toc237509330)

[Appendix 4 Decomposition of age-related changes in the mean number of diseases by disease group, ages 70–100, men and women, birth cohorts 1920–1922, Sweden	6](#_Toc237509331)

[Appendix 5 Disease coexistence pattern in men who survived two years or more at age 70, 80, 90 and 100, birth cohorts 1920 to 1922, Sweden	7](#_Toc237509332)

[Appendix 6 Disease coexistence pattern in women who survived two years or more at age 70, 80, 90 and 100, birth cohorts 1920 to 1922, Sweden.	8](#_Toc237509333)

[Appendix 7 Coexistence of specific diseases within the cardiovascular disease group in men at age 70, 80, 90 and 100, birth cohorts 1920 to 1922, Sweden.	9](#_Toc237509334)

[Appendix 8 Coexistence of specific diseases within the cardiovascular disease group in women at age 70, 80, 90 and 100, birth cohorts 1920 to 1922, Sweden.	10](#_Toc237509335)

[Appendix 9 Coexistence of specific diseases within the cardiovascular disease group in men who survived two years or more at age 70, 80, 90 and 100, birth cohorts 1920 to 1922, Sweden.	11](#_Toc237509336)

[Appendix 10 Coexistence of specific diseases within the cardiovascular disease group in women who survived two years or more at age 70, 80, 90 and 100, birth cohorts 1920 to 1922, Sweden.	12](#_Toc237509337)

# Appendix 1 Description of the registers included in current study

| **Variables** | **Total Population Register (TPR) [1]** | **National Patient Register[2, 3]** |
| --- | --- | --- |
| **Time periods** | A complete year-specific version of the TPR is available for each year since 1968. | Nationwide inpatient data have been available since 1987 and specialist outpatient data since 2001 and include essentially all specialist care in Sweden even if the provider is privately operated. |
| **Contents** | Birth, death, emigration, immigration to Sweden, migration within Sweden, marriage and divorce, country of birth, sex, citizenship | Patient-related data, including personal identify number (PIN), sex, age, county and municipality and parish; Data about caregiver (hospital/department); Administrative data, including admission date, discharge data, duration of admission etc; medical data, including diagnoses, primary and secondary diagnoses using ICD codes, procedures, psychiatric care. |
| **Unique identifiers** | Linking data in the population registers with other data is possible through the use of the personal identify number (PIN). The PIN consists of birthdate, birth number and a check digit. Details regarding PIN has been published elsewhere [4]. | For all Swedish residents, they have their unique personal identity number (PIN). Linking data in the population registers is possible through the unique PIN, which means, individuals included in current study have the same PIN in total population register (TPR) and the National Patient Register (NPR). |
| **Coverage** | High, virtually 100% of births and deaths, 95% of immigrations and 91% of emigrations in Sweden are reported to the Population Registers. | High, national coverage even if the provider is privately operated. [5] |
| **Quality** | High | High |
| **Data access and ethical aspects** | Data in the TPR are confidential and protected by the Secrecy Act (chapter 9, §4). This act stipulates that data become confidential on transmission to Statistics Sweden. Personal data from Statistics Sweden may still be delivered to researchers after appropriate approval by an Ethical Review Board and by an additional review by Statistics Sweden [6]. Statistics Sweden usually delivers de-identified data for research. | Data is accessible after appropriate ordering process (<https://bestalladata.socialstyrelsen.se/>) and Ethical approval. |

# Appendix 2 Swedish ICD9 and ICD10 codes used to identify diseases

| **Disease groups** | **Individual diseases** | **ICD−10** | **ICD−9** |
| --- | --- | --- | --- |
| Anemia | Anemia | D50-D64 | 280-285 |
| Cardiovascular diseases | Chronic rheumatic heart disease | I05**−**09 | 393-398 |
|  | Heart failure | I50 | 428 |
|  | Atrial fibrillation | I48 | 427D |
|  | Hypertension | I10−15 | 401-405 |
|  | Ischemic heart disease | I20−25 | 410-414 |
|  | Cerebrovascular diseases | I60−69, G45 | 430-438 |
|  | Cardiomyopathy | I42, I43 | 425 |
| Digestive disorders | Intestinal diverticula | K57 | 562 |
|  | Chronic liver disease | K70−77 | 571, 572, 573, 570 |
|  | Functional digestive disorders | K59 | 564F, 564G, 564H, 564W, 564X |
|  | Cholelithiasis | K80 | 574 |
| Endocrine diseases | Thyroid dysfunction | E00−07 | 240-246 |
|  | Diabetes | E10−14 | 259 |
| Malignancy | Malignant neoplasms | C00−97 | 140-208 |
| Neuropsychiatric diseases | Depression | F32; F33; F34; F38; F39 | 296B, 296W, 296X, 298A, 300E, 300X, 301B, 311 |
|  | Dementia | F00-F03, F05.1, G30, G31.1, G31.8A, G31.9 | 290, 294B, 331A-331C, 331X |
|  | Schizophrenia | F20−21 | 295A, 295B, 295C, 295D, 295F, 295G, 295W, 295X |
|  | Alcohol dependence syndrome | F10.2 | 303 |
| Musculoskeletal diseases | Osteoporosis | M80−81 | 733A, 733B |
|  | Osteoarthrosis | M15 | 715 |
|  | Rheumatoid arthritis | M05−06, M12.3 | 714A-714C, 714W, 714X, 719D |
|  | Crystal arthropathies | M10 | 274, 984X |
|  | Polymyalgia rheumatica | M35.3 | 725 |
| Neurosensorial diseases | Deafness, hearing impairments | H80, H90, H91.1, H91.3, H91.9 | 387, 389, 388A |
|  | Visual impairments or glaucoma | H54, H40 | 369, 365A-D, 365F-X |
|  | Migraine | G43 | 346 |
|  | Peripheral neuropathies | G50 | 350-359 |
|  | Parkinson disease | G20 | 332A |
|  | Epilepsy | G40 | 345,333C |
| Respiratory diseases | Chronic obstructive pulmonary diseases | J40−47 | 490-494, 496 |
|  | Pneumoconiosis | J60−66 | 500-505 |
| Urological disorders | Renal calculus | N20 | 592 |
|  | Prostate hypertrophy | N40 | 600 |
|  | Renal failure | N18, N19 | 585-586 |
| Additional added 5 conditions | | | |
| **Disease groups** | **Individual diseases** | **ICD−10** | **ICD−9** |
| Cardiovascular diseases | Peripheral vascular disease | I70, I71, I73.1, I73.8, I73.9, I77.1, I79.0, I79.2, K55 | 440, 441, 443B, 443W, 443X, 447B, 557 |
| Digestive disorders | Peptic ulcer disease | K25-K28 | 531-534 |
| Urological disorders | Urine incontinence | N39.3, N39.4, R32 | 625G, 788D |
| Digestive disorders | Fecal incontinence | R15 | 787G |
| Musculoskeletal diseases | Major Osteoporotic Fracture | S720-S722, S422, S525, S526, S220, S221, S320 | 820, 812A, 812B, 813E, 813F, 805C-F, 806C-F |

# Appendix 3 Mortality selection of the cohorts 1920-1922, Sweden.

# Appendix 4 Decomposition of age-related changes in the mean number of diseases by disease group, ages 70–100, men and women, birth cohorts 1920–1922, Sweden

Note: The figure shows the contribution of each disease group to the increase in the mean number of diseases over ages 70–100 years and within the age intervals 70–80, 80–90, and 90–100 years. For each interval, the change represents the absolute difference in the mean number of diseases between the beginning and the end of the interval (i.e., ages 70 and 100, 70 and 80, 80 and 90, and 90 and 100, respectively). Bars represent the absolute increase in the mean number of diseases attributable to each disease group, and percentages indicate the relative contribution of each disease group to the total increase within the corresponding age interval. The six disease groups contributing most to the total increase are shown separately; anemia, respiratory, endocrine, and neuropsychiatric diseases are combined as Other.

# Appendix 5 Disease coexistence pattern in men who survived two years or more at age 70, 80, 90 and 100, birth cohorts 1920 to 1922, Sweden

Notes: The x-axis shows the prevalence of the 45 most prevalent disease combinations, ranked in descending order. Disease combinations are represented by connected dots, with colors indicating the number of disease groups involved (single disease group: light yellow; 2 disease groups: yellow-green; 3: green-cyan; 4: cyan; 5: blue-cyan; 6: blue; 7: dark blue). Only disease combinations with a prevalence of at least 0.1% are shown. The panel "Prevalence of diseases (%)" displays the overall prevalence of each disease group, irrespective of whether it occurs alone or together with other disease groups. In contrast, the dot matrix and the corresponding "Prevalence of disease combinations (%)" columns display the prevalence of specific disease combinations.

**Example:** Among 70-year-old men surviving at least two years, 10.8% had cardiovascular disease (CVD), regardless of whether it occurred alone or in combination with other diseases. Of these, 7.8% had CVD only, while 0.5% had both CVD and urological disease, as indicated by the connected dots.

# Appendix 6 Disease coexistence pattern in women who survived two years or more at age 70, 80, 90 and 100, birth cohorts 1920 to 1922, Sweden.

Notes: The x-axis shows the prevalence of the 45 most prevalent disease combinations, ranked in descending order. Disease combinations are represented by connected dots, with colors indicating the number of disease groups involved (single disease group: light yellow; 2 disease groups: yellow-green; 3: green-cyan; 4: cyan; 5: blue-cyan; 6: blue; 7: dark blue). Only disease combinations with a prevalence of at least 0.1% are shown. The panel "Prevalence of diseases (%)" displays the overall prevalence of each disease group, irrespective of whether it occurs alone or together with other disease groups. In contrast, the dot matrix and the corresponding "Prevalence of disease combinations (%)" columns display the prevalence of specific disease combinations.

# Appendix 7 Coexistence of specific diseases within the cardiovascular disease group in men at age 70, 80, 90 and 100, birth cohorts 1920 to 1922, Sweden.

Notes: The x-axis shows the prevalence of the 45 most prevalent disease combinations, ranked in descending order. Disease combinations are represented by connected dots, with colors indicating the number of disease groups involved (single disease group: light yellow; 2 disease groups: yellow-green; 3: green-cyan; 4: cyan; 5: blue-cyan; 6: blue; 7: dark blue). Only disease combinations with a prevalence of at least 0.1% are shown. The panel "Prevalence of diseases (%)" displays the overall prevalence of each disease group, irrespective of whether it occurs alone or together with other disease groups. In contrast, the dot matrix and the corresponding "Prevalence of disease combinations (%)" columns display the prevalence of specific disease combinations.

# Appendix 8 Coexistence of specific diseases within the cardiovascular disease group in women at age 70, 80, 90 and 100, birth cohorts 1920 to 1922, Sweden.

Notes: The x-axis shows the prevalence of the 45 most prevalent disease combinations, ranked in descending order. Disease combinations are represented by connected dots, with colors indicating the number of disease groups involved (single disease group: light yellow; 2 disease groups: yellow-green; 3: green-cyan; 4: cyan; 5: blue-cyan; 6: blue; 7: dark blue). Only disease combinations with a prevalence of at least 0.1% are shown. The panel "Prevalence of diseases (%)" displays the overall prevalence of each disease group, irrespective of whether it occurs alone or together with other disease groups. In contrast, the dot matrix and the corresponding "Prevalence of disease combinations (%)" columns display the prevalence of specific disease combinations.

# Appendix 9 Coexistence of specific diseases within the cardiovascular disease group in men who survived two years or more at age 70, 80, 90 and 100, birth cohorts 1920 to 1922, Sweden.

Notes: The x-axis shows the prevalence of the 45 most prevalent disease combinations, ranked in descending order. Disease combinations are represented by connected dots, with colors indicating the number of disease groups involved (single disease group: light yellow; 2 disease groups: yellow-green; 3: green-cyan; 4: cyan; 5: blue-cyan; 6: blue; 7: dark blue). Only disease combinations with a prevalence of at least 0.1% are shown. The panel "Prevalence of diseases (%)" displays the overall prevalence of each disease group, irrespective of whether it occurs alone or together with other disease groups. In contrast, the dot matrix and the corresponding "Prevalence of disease combinations (%)" columns display the prevalence of specific disease combinations.

# Appendix 10 Coexistence of specific diseases within the cardiovascular disease group in women who survived two years or more at age 70, 80, 90 and 100, birth cohorts 1920 to 1922, Sweden.

Notes: The x-axis shows the prevalence of the 45 most prevalent disease combinations, ranked in descending order. Disease combinations are represented by connected dots, with colors indicating the number of disease groups involved (single disease group: light yellow; 2 disease groups: yellow-green; 3: green-cyan; 4: cyan; 5: blue-cyan; 6: blue; 7: dark blue). Only disease combinations with a prevalence of at least 0.1% are shown. The panel "Prevalence of diseases (%)" displays the overall prevalence of each disease group, irrespective of whether it occurs alone or together with other disease groups. In contrast, the dot matrix and the corresponding "Prevalence of disease combinations (%)" columns display the prevalence of specific disease combinations.

**References**

1.	Ludvigsson JF, Almqvist C, Bonamy A-KE, et al. Registers of the Swedish total population and their use in medical research. European Journal of Epidemiology. 2016 2016/02/01;31(2):125-36.

2.	Ludvigsson JF, Andersson E, Ekbom A, et al. External review and validation of the Swedish national inpatient register. BMC Public Health. 2011 Jun 9;11:450.

3.	Everhov ÅH, Frisell T, Osooli M, et al. Diagnostic accuracy in the Swedish national patient register: a review including diagnoses in the outpatient register. European Journal of Epidemiology. 2025 2025/03/27.

4.	Ludvigsson JF, Otterblad-Olausson P, Pettersson BU, et al. The Swedish personal identity number: possibilities and pitfalls in healthcare and medical research. Eur J Epidemiol. 2009;24(11):659-67.

5.	Ludvigsson JF, Bergman D, Lundgren CI, et al. The healthcare system in Sweden. Eur J Epidemiol. 2025 May 19.

6.	Ludvigsson JF, Håberg SE, Knudsen GP, et al. Ethical aspects of registry-based research in the Nordic countries. Clin Epidemiol. 2015;7:491-508.
